# Clinical oxygen enhancement ratio of tumors in carbon ion radiotherapy: the influence of local oxygenation changes

**DOI:** 10.1093/jrr/rru020

**Published:** 2014-04-11

**Authors:** Laura Antonovic, Emely Lindblom, Alexandru Dasu, Niels Bassler, Yoshiya Furusawa, Iuliana Toma-Dasu

**Affiliations:** 1 Department of Physics, Stockholm University, Stockholm, Sweden; 2 Department of Radiation Physics and Department of Medical and Health Sciences, Linköping University, Linköping, Sweden; 3 Department of Physics and Astronomy, Aarhus University, Aarhus, Denmark and Department of Experimental Clinical Oncology, Aarhus University Hospital, Aarhus, Denmark; 4 Next Generation Medical Physics Research Program and International Open Laboratories, National Institute of Radiological Sciences, Chiba 263–8555, Japan; 5 Department of Oncology and Pathology, Karolinska Institute, Stockholm, Sweden

**Keywords:** hypoxia, OER, TCP, RCR, carbon ion, fractionation, LOC

## Abstract

The effect of carbon ion radiotherapy on hypoxic tumors has recently been questioned because of low linear energy transfer (LET) values in the spread-out Bragg peak (SOBP). The aim of this study was to investigate the role of hypoxia and local oxygenation changes (LOCs) in fractionated carbon ion radiotherapy. Three-dimensional tumors with hypoxic subvolumes were simulated assuming interfraction LOCs. Different fractionations were applied using a clinically relevant treatment plan with a known LET distribution. The surviving fraction was calculated, taking oxygen tension, dose and LET into account, using the repairable–conditionally repairable (RCR) damage model with parameters for human salivary gland tumor cells. The clinical oxygen enhancement ratio (OER) was defined as the ratio of doses required for a tumor control probability of 50% for hypoxic and well-oxygenated tumors. The resulting OER was well above unity for all fractionations. For the hypoxic tumor, the tumor control probability was considerably higher if LOCs were assumed, rather than static oxygenation. The beneficial effect of LOCs increased with the number of fractions. However, for very low fraction doses, the improvement related to LOCs did not compensate for the increase in total dose required for tumor control. In conclusion, our results suggest that hypoxia can influence the outcome of carbon ion radiotherapy because of the non-negligible oxygen effect at the low LETs in the SOBP. However, if LOCs occur, a relatively high level of tumor control probability is achievable with a large range of fractionation schedules for tumors with hypoxic subvolumes, but both hyperfractionation and hypofractionation should be pursued with caution.

## INTRODUCTION

Hypoxia is a common cause of treatment failure in photon radiation therapy (RT), as cells that lack oxygen are more resistant to radiation than well-oxygenated cells [1]. The oxygen effect is often quantified in terms of the oxygen enhancement ratio (OER), which is the ratio of doses required to achieve the same biological effect under hypoxic and oxic conditions [1]. Typically, a certain level of cell survival serves as an endpoint. For anoxic cells irradiated with photons, the OER is about 3 for a surviving fraction (SF) of 10% [1]. For low linear energy transfer (LET) carbon ions, the OER is about the same as for photons. With increasing LET, the OER decreases and reaches unity at high dose-averaged LET-values of ∼500 keV/µm [2]. This means that the sensitivity of cells to intermediate- and high-LET carbon ion irradiation is less dependent on the oxygen tension compared with the sensitivity to photon irradiation. Carbon ion RT is thus assumed to result in better control of hypoxic tumors.

In the treatment of tumors with carbon ions, the Bragg peak (BP) is spread out in order to achieve better target coverage. A common range of the dose-averaged LET in the target is 30–80 keV/µm. This range yields an OER of 2–3 [2]. Therefore, the oxygen effect might have an impact on treatment outcome not only in photon RT, but also in carbon ion RT.

Current clinical routine treatment planning does not differentiate between hypoxic and well-oxygenated cells either in carbon ion or in photon RT. In photon RT, however, several different methods for taking tumor oxygenation into account have been proposed, explored and evaluated throughout the years [1, 3, 4]. Regarding carbon ion RT, only very few studies in which the oxygenation of the tumor is considered are available. Bassler *et al*. have proposed LET-painting, where hypoxic tumor subvolumes are irradiated with higher LET than well-oxygenated regions [5, 6]. Recently, Scifoni *et al*. presented a possible way to include tumor oxygen status in TRiP (TReatment planning for Particles), the standard carbon ion treatment planning system at GSI (Gesellschaft für Schwerionenforschung) [7]. Uniform biological effect is achieved within the tumor by an optimization approach that compensates for the increase in survival level in the hypoxic regions by increasing the dose to these, or redistributing the LET of the beams.

However, there are so far no publications investigating the effects of fractionation on treatment outcome for carbon ion RT of hypoxic tumors. Since hypoxia possibly plays an important role in carbon ion RT, another benefit of fractionated treatment, apart from the repair of normal tissue between fractions, might be the reoxygenation of hypoxic cells [8].

The mechanism of reoxygenation that is usually referred to in the literature is the slow reoxygenation of chronically hypoxic cells caused by tumor shrinkage [1]. However, another type of reoxygenation, resulting from changes in acute hypoxia between fractions, should also be considered [9–11]. In a recent study by Lindblom [12], the influence of such local oxygenation changes (LOCs) on the outcome of stereotactic body radiotherapy (SBRT) using photons was investigated. The study showed that extreme hypofractionation (using one single fraction) should be pursued with caution, since it might result in reduced local control as a result of the reduced possibilities of interfraction LOCs. The interplay between total dose, number of fractions, and thus the frequency of LOCs and their impact on the expected local control, should be of interest for carbon ion therapy studies as well, especially when considering the increased tendency towards hypofractionation in this form of RT [13–16].

Therefore, it is the aim of this study to theoretically investigate the influence of fractionation on the treatment outcome of tumors with heterogeneous oxygenations irradiated with carbon ions, assuming that LOCs take place between fractions.

## MATERIALS AND METHODS

### *In silico* tumor simulation and irradiation

Three-dimensional spherical tumors were simulated *in silico* with respect to oxygenation, number of clonogenic cells, and tumor size. Distributions of oxygen tension values within the tumor were based on a biologically relevant model of oxygen diffusion and consumption [17]. Distributions of intervessel distances, based on the experimental study by Konerding *et al.*, with different average values were used for the simulation of hypoxic (160 µm) and well-oxygenated regions (100 µm) [18]. Acute hypoxia was simulated by randomly shutting down a fraction of the blood vessel positions. The details of the model can be found in previous publications [17, 19, 20]. A uniform clonogenic cell density was assumed in all calculations.

**Fig. 1.**
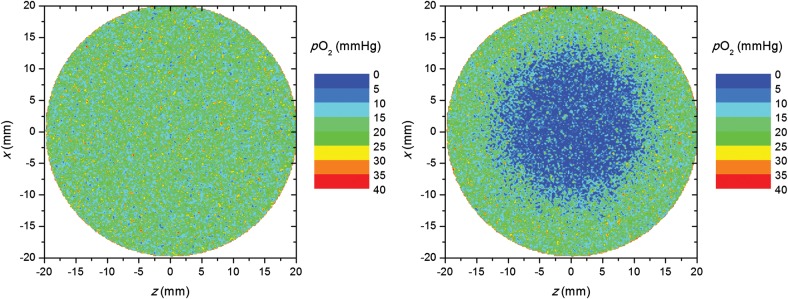
Oxygen-tension distributions in the well-oxygenated tumor (left) and in the tumor with a centrally positioned hypoxic island (right).

Two scenarios were considered for the tumor oxygenation: a well-oxygenated tumor, and a tumor containing a centrally positioned spherical hypoxic subvolume (Fig. 1). The diameters of the tumor and the hypoxic subvolume were 4.0 cm and 2.4 cm, respectively. The resulting hypoxic fractions (HFs) of the whole tumor and of the hypoxic subvolume were 16% and 63%, respectively, with a hypoxic threshold of 5 mmHg. The well-oxygenated tumor had a negligible hypoxic fraction (HF = 0.6%). Subsequent calculations were based on the distribution of oxygen tension values within the tumors.

**Fig. 2.**
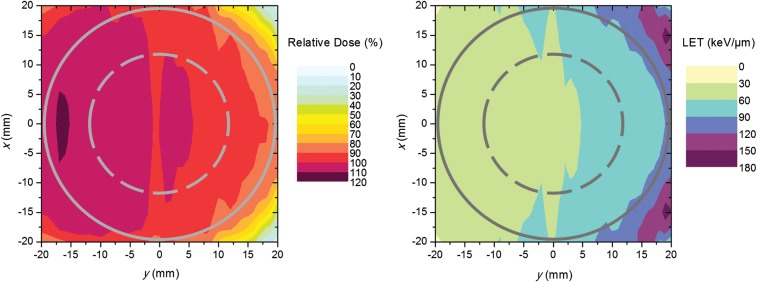
Cross sections of relative (physical) dose distribution (left) and dose-averaged LET distribution (right) in the clinical target volume. The circles indicate the clinical target volume position (full line) relative to the distributions as well as the center position of the hypoxic island (dashed line). The beam enters from the left.

A carbon ion treatment plan was made for the simulated tumor assuming a spherical planning target volume (PTV) with a diameter of 5 cm (0.5 cm PTV margin) centrally positioned in a large water phantom. One beam angle was used. The plan was optimized to give uniform biologically effective dose without taking the oxygenation of the tumor into account. The treatment planning system TRiP was used for the production of the plan [21]. Cross sections of the dose-averaged LET and absorbed dose distributions in the clinical target volume are presented in Fig. 2.

**Fig. 3.**
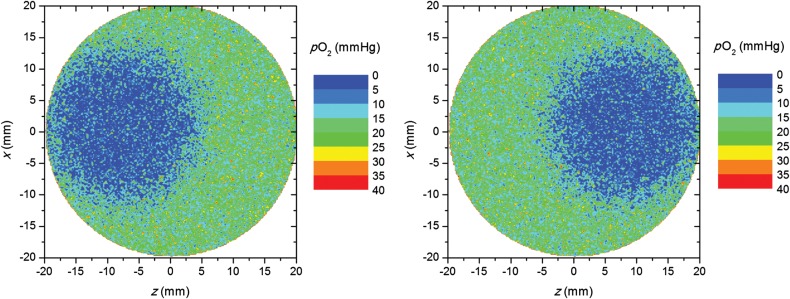
Oxygen-tension distributions in the tumors with the hypoxic island positioned ±8 mm off center, proximally (left) and distally (right). The beam enters from the left.

In order to investigate the influence of the hypoxic island position along the beam axis, and thus relative to the LET and dose distribution, two additional simulation geometries were considered, one with the hypoxic island positioned in the proximal part of the tumor relative to the beam entrance and one in the distal part (± 8 mm off center) (Fig. 3).

### Calculation of tumor control probability

The LET-parameterized repairable–conditionally repairable damage (RCR) cell-survival model was recently adapted to account for hypoxia by introducing a dose-modifying factor $\tilde{O}$ [22–24]. However, it can only be used to calculate the surviving fraction in either oxic or anoxic cell populations. In the present study, the model was further developed to take a continuous range of intermediate values of oxygen tension *p*O_2_ into account, by assuming the same dependence of $\tilde{O}$ on *p*O_2_ for carbon ions as the dependence of the OER on *p*O_2_ for photons [24, 25, 7]. The dose-modifying factor $\tilde{O}(L,p{\text{O}}_{2})$ taking the LET *L* and *p*O_2_ into account, is thus given by the following equations:

(1) $$\tilde{O}(\;L,p{\text{O}}_{2})=\tilde{O}(L)\cdot \frac{k+p{\text{O}}_{2}}{k+\tilde{O}(L)\cdot p{\text{O}}_{2}}$$

and

(2) $$\tilde{O}(L)={\tilde{O}}_{\min}+({\tilde{O}}_{\max}-{\tilde{O}}_{\min})e^{-{\left(\frac{L}{L_{\tilde{O}}}\right)}^{2}},$$

where ${\tilde{O}}_{\max}$ is the maximum dose-modifying factor in the absence of oxygen at minimal LET, ${\tilde{O}}_{\min}$ is the limit value at high LETs, $L_{\tilde{O}}$ is the LET where $\tilde{O}$ is reduced to 37% of its maximum value, and *k* is the value of the oxygen tension corresponding to the half effect, which was here set to 2.5 mmHg [7, 24, 26]. The surviving fraction *S* is subsequently described as a function of fraction dose *d*, LET and oxygen tension, as in equation 3:

(3) $$S(d,L,\;p{\text{O}}_{2})=e^{-a(L)d/\tilde{O}(L,\;pO_{2})}+b(L)d/\tilde{O}(L,\;p{\text{O}}_{2})e^{-c(L)d/\tilde{O}(L,\;pO_{2})},$$

where

(4) $$a\;(L)=a_{0}f\;(L)+a_{1}e^{-L/L_{n}},$$

(5) $$b\;(L)=b_{1}e^{-L/L_{n}},$$

(6) $$c\;(L)=c_{0}f\;(L)+c_{1}e^{-L/L_{n}},$$

(7) $$f\;(L)=\left(1-e^{-\frac{L}{L_{d}}}(1+L/L_{d})\right)\frac{L_{d}}{L}$$

and *a*_0_, *a*_1_, *b*_1_, *c*_0_, *c*_1_, *L*_n_ and *L*_d_ are free parameters depending on both the intrinsic radiosensitivity of the cells and the ion type [24].

In the present study, equations 2–7 were fitted to previously available and published cell-survival data from experiments of oxic and anoxic human salivary gland tumor (HSG) cells irradiated with carbon ions in the LET range of 22.5–501.5 keV/μm [2]. The dataset consisted of 19 and 20 survival curves, respectively, for the anoxic and well-oxygenated cells, with five to seven different doses for each LET and oxygen tension. The fitting was performed for the whole dataset simultaneously. Further details can be found in a previous publication [24]. The RCR parameters resulting from the fit were used in the subsequent calculations.

The choice of experimental data for this study is motivated by the use of the HSG cell line at the heavy-ion medical accelerator complex (HIMAC) at the National Institute of Radiological Sciences (NIRS) in Chiba and at the Gunma University, both in Japan, for planning biologic dose distributions in their clinical treatment planning systems [27].

The surviving fraction was calculated, voxel by voxel, depending on the oxygen tension, dose and LET of each voxel. The calculations were performed for different fraction sizes, the prescribed dose per fraction ranging from 0.7–10 Gy and the number of fractions, *n*, ranging from 3–36. LOCs were assumed to take place, meaning that the oxygen tension was assumed to change at the cellular level as a result of changes in acute hypoxia. This was achieved by randomly shutting down a fraction of vessels at each treatment fraction, while the average intervessel distance within each tumor region remained the same throughout the full course of treatment, leading to a redistribution of the *p*O_2_ values between voxels at each fraction [12, 17, 19, 20]. The overall geometry of the tumor with its hypoxic island, and the corresponding hypoxic fractions of the different regions, therefore, remained the same for all fractions (Fig. 1). For comparison, all simulations were also performed under the assumption that no local changes in oxygenation take place, i.e. that the oxygenation is static throughout the treatment.

The Poisson tumor control probability (TCP) dose–response model was used to predict the outcome from various combinations of fractionation schedules and oxygen distributions.

(8) $$TCP=\exp\left\{-\overset{N_{\mathrm{vox}}}{\underset{i=1}{\sum }}N_{i}\overset{n}{\underset{\;j=1}{\prod }}S_{i,j}(d_{i},L_{i},\;p{\text{O}}_{2i,j})\right\},$$

where *N*_vox_ is the total number of voxels, *N_i_, d_i_* and *L_i_* are the number of clonogenic cells, dose per fraction and LET in each voxel, and *S_i,j_* and *p*O_2 *i,j*_ are the surviving fraction and oxygen tension in voxel *i* at fraction *j*, respectively. In the case of static oxygenation, the surviving fraction of each cell remains constant during the treatment, and the equation above reduces to:

(9) $$TCP=\exp\left\{-\sum_{i=1}^{N_{\text{vox}}}N_{i}{\left[S_{i}\left(d_{i},L_{i},pO_{2i}\right)\right]}^{n}\right\}\cdot$$

The voxel size was 200 µm, and the total number of clonogenic cells in the tumor was set to 10^6^ in all simulations as the TCP values based on this number showed the best agreement with clinically reported values of local control [28]. The dose required to achieve a TCP of 50%, *D*_50_, was determined by fitting logit dose–response curves to the resulting TCP values [29].

In order to compare the effects of fractionation and LOCs on the treatment of well-oxygenated and hypoxic tumors, a clinical OER, *OER*_clin_, was defined as the ratio of *D*_50_ for well-oxygenated ($D_{50,\;\mathrm{ox}}$) and hypoxic ($D_{50,\;\mathrm{hypox}}$) tumors:

(10) $$OER_{\mathrm{clin}}=\frac{D_{50,\;\mathrm{hypox}}}{D_{50,\;\mathrm{ox}}}.$$

**Fig. 4.**
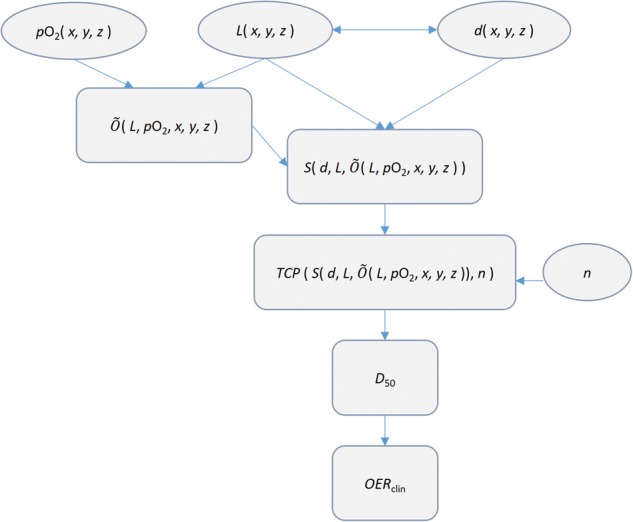
Flow chart describing the computation steps required to determine the clinical OER. Input variables are in elliptic boxes and calculated variables in rectangular boxes.

All computation steps required for determining the clinical OER are presented in a flow chart in Fig. 4.

## RESULTS

**Fig. 5.**
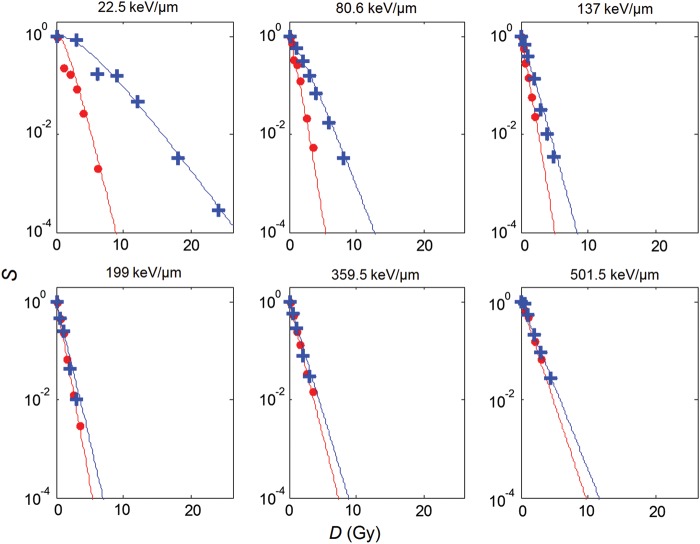
Oxic (red lines) and anoxic (blue lines) cell-survival curves resulting from the fit to experimental human salivary gland tumor oxic (dots) and hypoxic (crosses) cell-survival data using the LET-parameterized RCR cell-survival model modified to take oxygenation into account. The experimental data was obtained from from Furusawa *et al* [2].

**Fig. 6.**
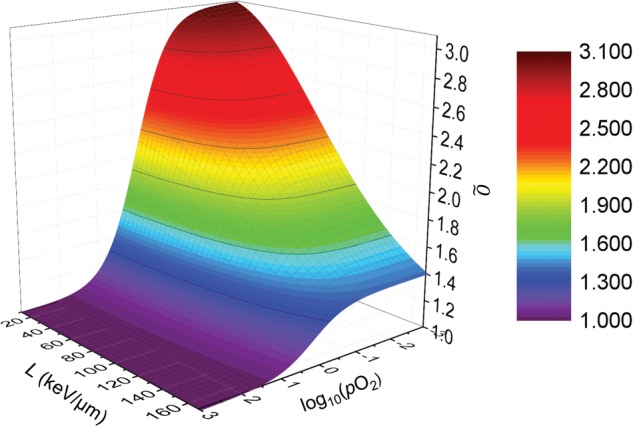
The dose-modifying factor $\tilde{O}$ as a function of LET and oxygen tension.

Figure 5 shows the results of the fit to the HSG cell-survival data from Furusawa *et al*. for a selection of six different LETs ranging from 22.5–501.5 keV/μm [2]. The fitting parameters$\;a_{0}$,$\;a_{1}$,$\;b_{1}$,$\;c_{0}$,$\;c_{1}$, $L_{n}$ and $L_{d}$ corresponding to the cell-survival curves shown in Fig. 5 are given in Table 1. The goodness-of-fit was assessed by calculating the root mean square deviation of the logarithm of the experimental cell survival from the analytical calculation, RMSD, which was 0.41, and the Pearson product-moment correlation coefficient, *r*^2^, which was 0.97. Figure 6 shows the resulting relationship between$\;\tilde{O}$, *L* and *p*O_2_.

**Table 1:** Parameters *a*_0_*, a*_1,_ *b*_1_*, c*_0_*, c*_1_*, L*_n_*, L*_d_*, Õ*_min_*, Õ*_max_ and *L_Õ_* resulting from the simultaneous fit to the hypoxic and anoxic HSG cell-survival data shown in Fig. 5

$a_{0}$	$a_{1}$	$b_{1}$	$c_{0}$	$c_{1}$	$L_{n}$	$L_{d}$	${\tilde{O}}_{\min}$	${\tilde{O}}_{\max}$	$L_{\tilde{O}}$
$\frac{1}{\text{Gy}}$	$\frac{1}{\text{Gy}}$	$\frac{1}{\text{Gy}}$	$\frac{1}{\text{Gy}}$	$\frac{1}{\text{Gy}}$	$\frac{\text{keV}}{\mu m}$	$\frac{\text{keV}}{\mu m}$			$\frac{\text{keV}}{\mu m}$
6.5	2.4	3.4	6.5	0.7	120	76	1.2	3.1	114

**Fig. 7.**
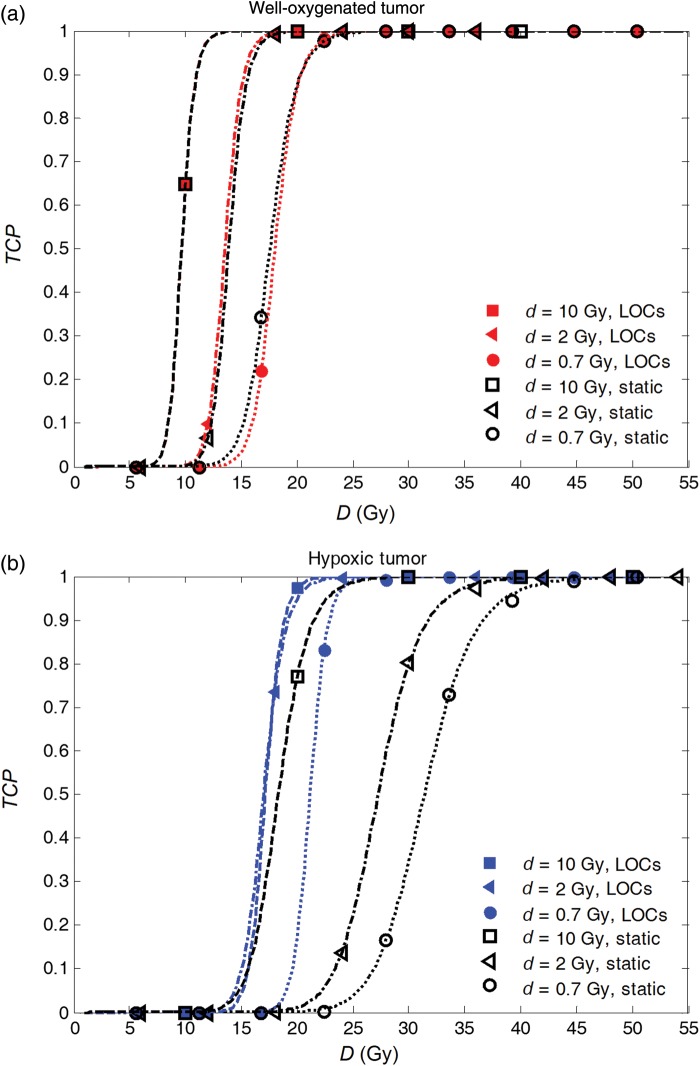
TCP as a function of total prescribed dose for fraction doses of 0.7 Gy, 2 Gy and 10 Gy for: **(a**) a well-oxygenated tumor and (**b**) a hypoxic tumor.

Figure 7a and b show dose–response curves in terms of TCP as a function of total prescribed dose *D* for the well-oxygenated and for the hypoxic tumors, respectively, assuming that the total dose is delivered in fractions of 0.7 Gy (= 2.1 GyE), 2 Gy (= 6 GyE) and 10 Gy (= 30 GyE). Curves resulting from simulations assuming static oxygenation and LOCs are shown in the same panels. As expected, there are negligible differences for the well-oxygenated tumor between the curves corresponding to the static oxygenation case and assuming LOCs (Fig. 7a).

For the hypoxic tumor, on the other hand, and for low fraction doses (*d* = 0.7 Gy and *d* = 2 Gy) there are large differences in the dose required to control the tumor between the case when LOCs take place and the static oxygenation case (Fig. 7b). The dose required to achieve a TCP of 50%, *D*_50_, is ∼10 Gy higher if the oxygenation is static compared with the case when LOCs are allowed to occur. For the highest fraction dose of 10 Gy, the difference in the resulting *D*_50_ between the two oxygen dynamics cases is much smaller.

For the hypoxic tumor, it should be noted that for *d* = 10 Gy and *d* = 2 Gy, the dose–response curves assuming LOCs almost coincide. For the lowest fraction dose (0.7 Gy), *D*_50_ is ∼5 Gy higher than for the two higher fraction doses. This means that if LOCs are assumed to take place, the total dose required to control the tumor is almost independent of the size of the fraction dose if the treatment is delivered with doses per fraction between 2 and 10 Gy. This observation is further illustrated by the comparison of the values of *D*_50_ that all lie in the interval between 15.5 Gy (*d* = 5 Gy) and 17.2 Gy (*d* = 10 Gy) for the fraction doses of 2 Gy, 3 Gy, 4 Gy, 5 Gy, 6 Gy, 8 Gy and 10 Gy. For the lower fraction doses of 1 Gy and 0.7 Gy, *D*_50_ is 19.2 Gy and 21.1 Gy, respectively, i.e. 4–5 Gy higher than for the higher fraction doses, as mentioned above.

Another aspect that could also be noted in Fig. 7b is the change in the slope of the dose–response curves when different scenarios are assumed with respect to the oxygenation of the tumor. The shallow curve obtained for *d* = 0.7 Gy and static oxygenation has a normalized slope of the curve of γ = 3.5. The curve is steeper when LOCs are assumed (γ = 7). The trend is the same for the other fraction doses, and this is explained by the fact that the LOCs lead to an overall increase in the homogeneity in the response of the cells, thus making the dose–response curve steeper, while the shallower curve reflects the larger heterogeneity in the response of the cells with different oxygenations kept constant during the course of the treatment [30].

**Fig. 8.**
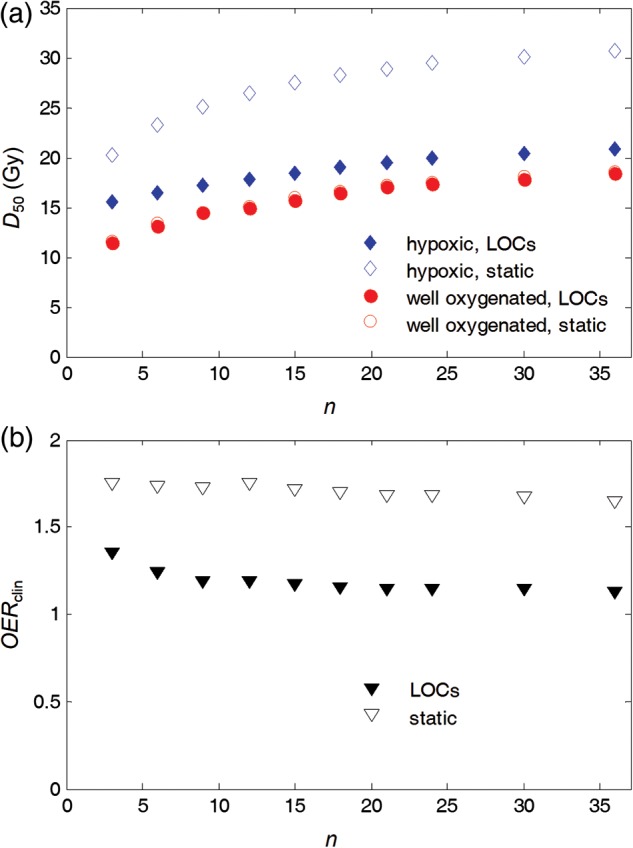
(**a**) Total dose required to achieve a TCP of 50%, *D*_50_, for different numbers of fractions for well-oxygenated and hypoxic tumors, with local oxygenation changes (LOCs) and with static oxygenation. (**b**) Clinical OER for different numbers of fractions assuming LOCs or static oxygenation.

In Fig. 8a, the relationship between *D*_50_ and the number of fractions *n* is shown. As expected, when the dose per fraction is lowered and the number of fractions is increased, *D*_50_ increases, owing to the repair of sublethal damages [31, 32]. However, the trend in the variation of *D*_50_ with the number of fractions has to be analyzed in conjunction with the difference in *D*_50_ between well-oxygenated and hypoxic tumors. Figure 8b shows the dependence of the clinical OER, defined as the ratio of *D*_50_ for hypoxic and well-oxygenated tumors, on the number of fractions. For the static oxygenation case, *OER*_clin_ lies in the range 1.65–1.75 for all numbers of fractions investigated in this study (3–36). Assuming LOCs, *OER*_clin_ is <1.2 for all numbers of fractions between nine and 36. In this range, the variation of *OER*_clin_ with *n* is low, with the difference in *OER*_clin_ between two adjacent points being ≤1.8%. If these values are compared with the corresponding ones for extremely hypofractionated schedules, the value of *OER*_clin_ increases to 1.36 for *n* = 3, and the difference between two adjacent points becomes considerably larger, namely as large as 18%.

In order to investigate the effect of the position of the hypoxic island in the beam direction (*z*-axis) on the TCP, dose–response curves were determined for a fraction dose of 1 Gy for the case when the hypoxic island is located close to the proximal edge of the tumor relative to the beam entrance or close to the distal edge, as illustrated in Fig. 3. The average values of LET *L* and the dose modifying factor $\tilde{O}$ within the hypoxic island were also calculated for the three different positions and are presented in Table 2 together with the resulting absolute values of *D*_50_. When shifting the island position 8 mm off center closer to the proximal edge, where the average LET is lower than in the center of the tumor, *D*_50_ increased by 8.8% when assuming LOCs. When shifting the island 8 mm closer to the distal edge, where the LET is higher, *D*_50,_ instead, dropped by 6.4% compared with the center position. For the static oxygenation case, *D*_50_ increased by 9.5% and decreased by 12.0%, respectively. In the *x* or *y* directions, on the other hand, the changes in *D*_50_ were ≤2.2% for any positional shifts of 8 mm relative to the center position. The explanation for the decrease in *D*_50_ when moving the hypoxic island closer to the distal edge is the increase in LET (and thus the decrease in $\tilde{O}$) in this region (Fig. 2), as described by equations 1–9. The opposite holds when the hypoxic island is positioned closer to the proximal edge.

**Table 2:** Values of the total dose required to achieve a TCP of 50%, *D*_50_, of the average LET *L* and of the average dose-modifying factor *Õ* of the hypoxic tumor with *d* = 1 Gy

Position of hypoxic island	Average *L* (keV/µm)	Average *Õ*	*D*_50_ (Gy) Static	*D*_50_ (Gy) LOCs
**Proximal edge**	50.5	1.46	34	20
**Center**	56.9	1.45	30	19
**Distal edge**	67.8	1.42	27	18

## DISCUSSION

One of the most quoted advantages of carbon ion radiotherapy is the potential ability to control hypoxic tumors, as the oxygen effect is weaker for this type of radiation compared with conventional photon RT [33, 34]. However, this classic paradigm has recently been questioned, since the reported dose-averaged LET-values in the SOBP that covers a typical target are lower than the dose-averaged LET-values in the pristine Bragg peaks of the *in vitro* experiments that have been used to demonstrate the efficacy of carbon ions in killing anoxic cells [5–7, 33]. The increased awareness of the role that oxygen might play in carbon ion RT has led to propositions of LET-painting for counteracting hypoxia-related radioresistance and to the implementation of radiobiological models in a treatment planning system aiming to adapt the prescribed dose and beam components according to the oxygenation of the tumor [5–7].

Nonetheless, clinical experience in the use of carbon ion radiation therapy is growing, and it is demonstrating good outcomes for a large variety of tumor types and sites (including hypoxic tumors) [28, 35]. This indicates that, besides tumor oxygenation, dose and LET, further related aspects should be considered when analyzing clinical data or when predicting the outcome of various clinical schedules.

The present study adds to the previous efforts to include tumor oxygenation at the planning stage of treatment by analyzing the impact of hypoxia and reoxygenation between fractions on the predicted response of tumors with heterogeneous oxygenations. Our results are in agreement with the results of other theoretical studies indicating that a considerable increase in dose would be required to counteract the impact of hypoxic areas if oxygenation is static [5–7]. However, our results highlight the importance of also considering the dynamics of hypoxia.

The results of the present study clearly show that the dose needed to control hypoxic tumors assuming LOCs is considerably lower than if oxygenation is static, as is indicated by the difference in *OER*_clin_ for the two cases. As expected, the magnitude of the improvement in TCP depends on the number of fractions (Figs 7 and 8). The improvement decreases for extremely hypofractionated schedules, simply because fewer interfraction oxygenation changes can take place. The *OER*_clin_ drops rapidly between three and nine fractions, as shown in Fig. 8. If more than nine fractions are employed, the *OER*_clin_ is rather independent of *n*. Lengthy schedules would, therefore, not bring a further improvement in *OER*_clin_, but they could increase the risk of accelerated proliferation taking place [36].

Another way to analyze the impact of fractionation is to investigate the dependence of the total dose required for tumor control on the size of the dose per fraction. It is well known that the total dose required for tumor control decreases with increasing size of the fraction dose. For well-oxygenated tumors, this is illustrated in Fig. 7a, which clearly shows the differences in dose–response for different fraction doses. This is also the case for hypoxic tumors with static oxygenation (Fig. 7b). However, for hypoxic tumors in which LOCs take place between fractions, the total dose required for tumor control is rather constant for a wide range of fraction doses (2–10 Gy). This means that the expected increase in total dose required for tumor control when going from high to low fraction doses is balanced by the beneficial effects of LOCs over a wide range of fraction doses. The expected increase in total dose due to repair between fractions is only found at fraction doses <2 Gy.

In a clinical study by Nakano *et al*., the carbon ion treatment outcome for hypoxic tumors was shown to be equally as good as the treatment outcome for well-oxygenated tumors [35]. The treatment was delivered in 24 fractions, which means that LOCs were allowed to occur. The high number of fractions might thus explain the good results for the hypoxic tumors, although the fraction doses employed in that study (0.7–1 Gy) are just below the threshold of what is efficient with respect to tumor control based on our study. The results of the study by Nakano *et al*. might, therefore, have been further improved, for both hypoxic and well-oxygenated tumors, if fraction doses were slightly increased.

Even though we have identified thresholds regarding both hypo- and hyperfractionation for the specific tumors of our study, no optimal fractionation schedule can be recommended in general for carbon ion radiotherapy of the wide variety of tumors encountered in the clinic. Our study shows that the position of the hypoxic island will have some impact on the dose required for tumor control, especially in the direction of the beam axis along which the average LET changes the most. It is well known that high-LET radiation is more efficient in killing both well-oxygenated and hypoxic cells compared with low-LET radiation [1]. As mentioned in the introduction, the protective effect of hypoxia is also weaker at high LETs. The increased effectiveness of the relatively high LET-values in the distal part of the tumor is therefore expected to be more pronounced for hypoxic than for well-oxygenated cells. Our results, showing that *D*_50_ decreases towards the distal part of the tumor, are therefore not surprising. The contribution of our results is, rather, that they give a quantitative estimate of the noted dependencies. Our results also show that the sensitivity of *D*_50_ to the position of the island is lower if LOCs are assumed. Other aspects that we did not fully explore, such as the size of the hypoxic island and the fraction of hypoxic cells, are also likely to influence the benefit of fractionating treatment with regard to LOCs.

The simulations in this study have been based on experimental observations regarding the spatial and temporal distribution of oxygen in the absence of tumor shrinkage [9–11]. These observations strongly support our assumption that LOCs do take place between fractions. Ljungkvist *et al*. demonstrated, on a microscopic scale, the heterogeneous distribution of well-oxygenated and hypoxic areas in tumor xenografts [9]. Furthermore, using a dual-staining technique, the same research group showed that the LOCs take place within several minutes, thereby demonstrating that the spatial distribution of oxygen in tumors is unlikely to be the same for two consecutive fractions separated by a few hours [10]. These observations were recently confirmed by Redler *et al*., who used electron paramagnetic resonance measurements to show that local oxygenation in tumors fluctuates within several tens of minutes [11]. With the background of these experimental observations, it appears probable that our results for the LOCs case are representative of clinical tumors.

Generally, it is thought that cells suffering from the two different types of hypoxia, chronic and acute, have the same induced radioresistance, but recently it has been suggested that the radiosensitivity may be modulated by the metabolic energy status of the cells [37]. Based on experimental data, it has also been suggested that chronically hypoxic cells might be more sensitive than acutely hypoxic cells [38–40]. The present study, however, does not take into account the difference in sensitivity between the two types of hypoxic cells, since they cannot yet be distinguished *in vivo*.

Despite the possible improvements in TCP if LOCs are allowed for, it is important to note that hypoxia seems to be an important factor in modulating clinical outcome, as our results show that the *OER*_clin_ is well above unity both for the static as well as for the dynamic oxygenation cases and for all fractionations. Unless slow reoxygenation of chronically hypoxic cells (caused by tumor shrinkage during the course of treatment) is assumed to take place on top of the local changes in acute hypoxia, it might therefore be beneficial to consider the oxygenation status of tumors at the planning stage of carbon ion RT.

## CONCLUSION

In conclusion, our results suggest that hypoxic tumors could be controlled to almost the same degree as well-oxygenated tumors, if LOCs were allowed to take place between fractions. With regard to tumor control, extreme hypofractionation should thus be avoided in order to allow for interfraction LOCs, while extreme hyperfractionation should be employed only with great care, as it increases the total dose required for tumor control and might also lead to accelerated proliferation if overall treatment time is prolonged.

## FUNDING

This work was supported by CIRRO—The Lundbeck Foundation Center for Interventional Research in Radiation Oncology, The Danish Council for Strategic Research, Novo Nordisk Fonden and by the Danish Cancer Society Project [ID#DP08023 to N.B.] and Cancer Research Funds of Radiumhemmet [124052 to E.L.]. Funding to pay the Open Access publication charges for this article was provided by Radiumhemmet.
